# Cooperative care slows offspring telomere attrition and buffers growth against variable rainfall in a wild Kalahari bird

**DOI:** 10.1098/rstb.2025.0325

**Published:** 2026-11-12

**Authors:** Pablo Capilla-Lasheras, Emma M. Wood, Antony Brown, Andrew J. Young

**Affiliations:** ^1^ Centre for Ecology and Conservation, University of Exeter, Penryn, Cornwall, UK; ^2^ Bird Migration Unit, Swiss Ornithological Institute, Sempach, Switzerland; ^3^ Estación Biológica de Doñana, Seville, Spain

**Keywords:** altruism, senescence, development, cooperation, environmental buffering, ageing, growth, cooperative breeding, helping

## Abstract

Recent studies suggest that early-life competition can compromise somatic integrity and exacerbate late-life declines in performance (senescence). However, the potentially beneficial effects for somatic integrity of early-life receipt of cooperation remain poorly understood. Here we investigate how the receipt of cooperative alloparental care impacts developmental telomere dynamics (a biomarker of somatic integrity) and growth in cooperatively breeding white-browed sparrow weavers, *Plocepasser mahali*. In sparrow-weaver groups, a single breeding pair monopolize reproduction, non-breeding helpers assist with nestling provisioning, and both helper number and rainfall (in this semi-arid environment) positively predict nestling provisioning rate. Accordingly, we show that nestlings reared by more helpers enjoy (i) slower telomere attrition rates (known to predict downstream survival in this species), and (ii) higher growth rates, which are also buffered against variation in rainfall (owing to stronger beneficial effects of helpers in dry conditions). These relationships cannot be readily attributed to correlated variation in maternal egg investment or among-territory variation in food availability. These findings provide rare evidence that alloparental care can bolster the somatic integrity of offspring, highlighting the potential for downstream effects on their senescence trajectories. Our findings have implications too for understanding the resilience of cooperative species in increasingly unpredictable rainfall regimes.

This article is part of the theme issue ‘Developmental circuits in ageing’.

## 1. Introduction

The early-life social environment can profoundly affect downstream adult performance and senescence trajectories [1–7]. These effects are hypothesized to arise in part via effects of the social environment on early-life somatic integrity (the extent to which somatic tissues are free from biomolecular damage and errors) that, in turn, impact later-life performance [8–13]. For example, numerous studies using telomere length dynamics as a biomarker of somatic integrity suggest that early-life exposure to competition can negatively impact the somatic integrity of developing young [10–15]. However, it is less clear whether early-life exposure to cooperative behaviour can positively impact the somatic integrity of developing young [13,16–18]. Cooperatively breeding societies, in which ‘helpers’ assist parents with offspring care [19,20], provide a useful testing ground for this idea. The cooperative contributions of helpers commonly increase the rate at which offspring are provisioned [20–22] and could thereby alleviate resource-based constraints on the competing developmental demands of growth and somatic maintenance [23,24]. Positive effects of helpers on offspring growth and early-life survival are widely reported (e.g. [20,22,25]), but there is evidence too of beneficial downstream effects of helping on the later-life performance of offspring in adulthood (e.g. [1,2,7,26]), highlighting the possibility that helpers improve the somatic integrity of developing young. With the advent of molecular biomarkers of somatic integrity, it has now become possible to investigate whether helping does have cryptic beneficial effects on offspring somatic integrity [13,16–18].

Attempts to study somatic integrity and its maintenance have been revolutionized in recent years by the discovery that telomere lengths and their temporal dynamics can serve as biomarkers of somatic integrity [8,9,27–31]. Telomeres are ribonucleoprotein structures that cap the ends of linear chromosomes, buffering coding DNA from the erosion that occurs during cellular replication [27,32,33]. In the absence of telomere repair mechanisms, telomeres shorten with each cell division and under exposure to oxidative stress [32,33], highlighting the potential for telomere attrition to serve as a biomarker of accumulated historical exposure to oxidative damage [27–30,32–34]. Two widely reported findings across natural populations of birds and mammals suggest that telomere length and/or its temporal dynamics can indeed serve as an effective biomarker of somatic integrity in such taxa [28–30,33]. First, mean telomere lengths in the somatic tissues of birds and mammals tend, on average, to decrease with advancing age [31] (though patterns can vary markedly, including evidence suggestive of bouts of lengthening [35]), attrition that appears to accelerate under exposure to diverse stressors [34]. Second, shorter mean telomere lengths and/or higher rates of telomere attrition are commonly predictive of poorer downstream survival [8,9,36].

At least two mechanisms, acting in isolation or concert, could explain why telomere length appears to act as a biomarker of somatic integrity in natural animal populations [27–30,37]. First, short telomeres could play a causal role in undermining tissue function, as the accumulation of critically short telomeres within a cell can trigger apoptosis or cellular senescence, an irreversible state of cell cycle arrest now causally implicated in tissue dysfunction and (thereby) organismal senescence in laboratory models [38,39]. However, to what extent critically short telomeres are a major proximate cause of age-related declines in organismal performance in natural animal populations is not yet clear [10,29,30,37]. Second, even if short telomeres played no causal role in tissue dysfunction in nature, the mechanisms that accelerate telomere shortening (including cellular proliferation and oxidative damage) have the potential to cause correlated damage to other biomolecules that could themselves undermine tissue and organismal performance [27,32,34]. Telomere attrition could thereby serve as an effective biomarker of this accumulated damage to other consequential structures [27,29,30,32]. Indeed, several studies have found that telomere attrition rate is more strongly predictive of downstream organismal mortality than having shorter mean telomere lengths (e.g. [10,37,40] though see also [41,42]), a finding that aligns with expectation if telomere attrition rate acts as a biomarker of correlated damage to other structures whose deficits are more causal than telomere length *per se* in undermining tissue performance [10,37,40]. The strongest insights about somatic integrity and its maintenance during a particular life period may therefore stem from longitudinal studies that use repeated measures of telomere length across the focal period to study within-individual telomere attrition rates during that period, independent of any pre-existing inter-individual differences in mean telomere length (which could arise in part via mechanisms unrelated to somatic integrity, such as genetic and epigenetic variation in any ‘initial’ telomere length set during early development [10,37,40–44]).

Growing evidence that early-life exposure to competition can accelerate telomere attrition suggests that such competition can undermine somatic maintenance [10–15] and highlights the potential for early-life exposure to cooperation, by contrast, to bolster somatic maintenance. For example, both experimental increases in brood size and the generation of competitive disadvantages within a brood have been shown to accelerate telomere attrition in nestlings [10–12,15]. Intensified competition could accelerate telomere attrition via a range of mechanisms, including a reduction in resource availability for the competing demands of growth and somatic maintenance [14,23,24] and any associated stress responses themselves further accelerating telomere attrition [34,45]. These same pathways provide credible mechanisms by which cooperative helping behaviour could ameliorate developmental telomere attrition among offspring, in cooperatively breeding species in which helpers increase the overall rate at which offspring are fed. However, compelling evidence that helping behaviour does ameliorate developmental telomere attrition in offspring remains elusive (see [46] for effects of either helping or cobreeding on the somatic integrity of mothers). In cooperatively breeding meerkats, *Suricata suricatta*, and purple-crowned fairy wrens, *Malurus coronatus*, for example, helpers appear to increase the rates at which offspring are fed and survive but do not appear to leave them with longer telomeres [7,13,17,25,47]. In superb fairy wrens, *Malurus cyaneus*, offspring telomere lengths are also unrelated to helper numbers [18], though in this species the additive contributions of helpers to nestling care could be offset by mothers reducing their prenatal investment in the egg when helped [48]. Just one study has revealed some support, albeit mixed, for the hypothesis that helping behaviour can improve offspring somatic integrity. In sociable weavers, *Philetairus socius*, hatchlings born into groups with no helpers and cross-fostered into groups with helpers showed longer telomeres at 9 days of age than controls (cross-fostered into groups with no helpers) [16]. However, this difference was no longer evident at 17 days of age, and the results for offspring cross-fostered from groups with helpers did not conform with expectations [16]. Together, these findings may indicate that helpers rarely do impact the somatic integrity of offspring. Notably though, all of these studies assessed effects of helper numbers on offspring telomere length, rather than within-individual rates of telomere attrition during the helping period. It is therefore conceivable that pre-existing inter-individual differences in telomere length (potentially unrelated to somatic integrity; see above) rendered helper effects on telomere attrition challenging to detect in analyses of single measures of offspring telomere length.

Here we investigate the effects of helping behaviour in cooperatively breeding white-browed sparrow weaver, *Plocepasser mahali*, societies on the growth and longitudinal telomere length dynamics of offspring during the postnatal-dependent period (when helping occurs). White-browed sparrow weavers live in year-round territorial groups throughout the semi-arid regions of sub-Saharan Africa [49,50]. They are rain-dependent breeders, rearing broods at any time during the southern summer (most clutches are laid October–April) in response to recent rainfall, the key abiotic driver of reproductive success in this species [51–53]. Within each social group, a single dominant male and female monopolize reproduction, and up to 10 non-breeding subordinates of both sexes help to feed their nestlings (hereafter ‘helping’; [50,51,54,55]). Clutches are small, typically just two eggs (range 1–3), and do not vary in size with helper numbers [53]. As subordinates (hereafter ‘helpers’) are typically offspring from previous broods that have delayed dispersal from their natal group, they are typically helping to rear their parents’ young (to whom they are therefore closely related [50,55]) and thus would gain indirect fitness benefits from helping if it increased the fitness of the parents (the dominants) or the offspring [50,54,55]. Helping does appear to improve parental fitness. When mothers have more helpers, they reduce their nestling provisioning rates [53] and enjoy both higher annual survival [56] and lower rainfall-induced variance in reproductive success (an effect that may enhance parental fitness via bet-hedging mechanisms in this unpredictable rainfall regime [51]). Whether helping enhances offspring fitness is less clear. Helper numbers positively predict the rates at which offspring are fed but appear to have no effect, on average, on offspring survival to fledging [51]. This is because helper numbers positively predict offspring survival to fledging in dry conditions (when food is scarce and nestling starvation is common) but negatively predict offspring survival in wetter conditions (perhaps because their positive effect on nest visitation rate leaves helped nests more conspicuous to predators), resulting in no overall effect of helpers on the mean rate of survival to fledging once one integrates across rainfall conditions [51]. However, helping could still improve offspring fitness by improving the quality of those offspring that do survive to fledging. By increasing the rate at which offspring are fed, helpers could alleviate resource constraints on the competing demands of growth and somatic maintenance, leading to enhanced growth and/or somatic integrity among dependent young.

First, we provide context for our study by showing that helper numbers and recent rainfall are the key environmental drivers of the rates at which broods of nestlings are fed. Second, we investigate the effects of natural variation in helper number and rainfall on postnatal growth (assessed as nestling mass at 7–9 days of age and then again post-fledging). Given their positive effects on provisioning rate, we predict positive effects of both helper number and recent rainfall on nestling growth and that these should be evident by 8 days of age. Similar effects may also be evident in offspring body mass after fledgling, unless initially lighter (slower growing) offspring catch up on growth by subsequently growing faster or for longer [57,58]. As the nutritional needs of offspring may be adequately met by either high helper numbers or high rainfall, helper number and rainfall may also have interactive effects on growth, with helpers having their most positive effects on growth in dry conditions (as we have previously detected for nestling survival to fledging [51]). Third, we investigate the effects of helper numbers and rainfall on the within-individual rate of change in mean whole blood telomere length during the postnatal-dependent period, using offspring blood samples collected longitudinally from 4 to 35 days post-hatching (fledging typically occurs around 20–25 days post-hatching). Here we made the same predictions as for growth; both helper number and rainfall should have positive (and potentially interactive) effects on the rate of change in mean telomere length during development (i.e. negative effects on developmental telomere attrition rate), given their positive effects on the rate at which offspring are fed. Given the potential for a trade-off between growth and self-maintenance, we also note the possibility that, if helpers or rainfall markedly increase the growth rate, this could instead lead to accelerated telomere erosion among offspring reared in these contexts [23,59]. Factors that increase the within-individual rate of change in mean whole blood telomere length during postnatal development (i.e. decrease telomere attrition rate) are generally expected to yield downstream fitness benefits to offspring arising from improved somatic integrity [8–10,37]. Indeed, in sparrow weavers, the within-individual rate of change in mean whole blood telomere length during postnatal development (the metric studied here) positively predicts the downstream survival of offspring to adulthood [37], while absolute mean telomere length does not [37].

## 2. Methods

### (a) Study population and field methods

Our study population comprises approximately 40 cooperative groups of white-browed sparrow weavers, defending year-round territories in an approximately 1.5 km^2^ area of Tswalu Kalahari Reserve, South Africa (27°16′ S, 22°25′ E). White-browed sparrow weavers in this population may breed at any time from September through to May (the southern summer), depending on the timing of unpredictable summer rainfall [51,52]. Each bird within our study population is fitted with a metal ring and three colour rings, providing a unique ring combination for identification in the field (SAFRING licence 1444). From around six months of age, birds of the focal subspecies (*P. mahali mahali*) can be readily sexed in the field by their bill colour; males have a dark brown bill, while females have a paler grey-to-pink bill [50]. Birds that did not survive to this age were sexed using molecular techniques [50]. All protocols were approved by the Ethics Committees of the University of Pretoria and complied with regulations stipulated in the Association for the Study of Animal Behaviour (ASAB) Guidelines for Use of Animals in Research.

Sparrow-weaver groups were easily monitored and distinguished in the field, as all group members foraged together, engaged in cooperative provisioning, weaving, vigilance and territorial defence and roosted within a single tree or cluster of trees close to the centre of their territory [49,60–62]. Dominance status and social group compositions were determined via regular (at least twice weekly) visits to the focal groups throughout each field season, with birds being identified based on their colour-ring combination. Social dominance was assigned based on the monitoring of key dominance-related behaviours: the dominant pair routinely displace other group members and produce synchronized duet song, the dominant female is the sole incubator, and the dominant male consistently produces dawn song during breeding periods [50,63,64]. Birds were also routinely caught while roosting within their group's territory at night, and this information also contributed to group composition assessments. Group compositions were typically very stable over time, with group members residing within the same social group for many months to many years at a time, with group membership not fluctuating according to short-term changes in environmental conditions [53]. Birds were considered to belong to a social group when they were consistently observed to forage with that group and roost in trees within the group's territory [50]. Start and end dates for group membership were calculated as follows: the start of group membership was deemed to be the date midway between a bird last being known to be absent from the group and first being seen as accepted in the group; the end of the group membership was deemed to be the date midway between when they were last known to be present and first confidently recorded as absent. For every breeding attempt in our analyses, we used these group compositions to calculate the number of helpers that were available to help the dominant pair rear the focal brood. All subordinate group members over the age of six months were considered helpers, as analyses of helper contributions suggest that subordinates less than six months old contribute little to nestling provisioning [49,55].

### (b) Breeding attempt monitoring

The breeding status of each group was determined by monitoring the contents of all woven nest structures within each group's territory using an endoscope at least every other day during the breeding season. When one or more eggs were newly detected, the active nest was visited daily in the afternoon until no new eggs were detected; the birds lay one egg per day in the morning and typically lay clutches of two eggs (range 1–3 [50]). To determine hatch dates, daily monitoring of the active nest resumed 14 days after the detection of the first egg (as incubation lasts 14–19 days [50]). This method yielded accurate information on the day on which the first nestling in each clutch hatched, which was termed ‘day 1’ of the nestling period for the focal breeding attempt. Upon first discovery, eggs were measured (length and width, for the calculation of egg volume [53]), and nestlings were weighed to the closest 0.01 g (Durascale 100; MyWeigh, Phoenix, AZ) and assigned an individually identifiable mark (trimming the downy feathers on the left side, top or right side of their head) to allow their longitudinal monitoring until ringing on day 13 (SAFRING licence 1444). Nests were checked for nestling survival on days 5, 9, 13 and 17 of the nestling period (as nestlings can fledge soon after day 17, subsequent nest visits were avoided).

### (c) Provisioning rate data collection

We monitored the rates at which all group members provisioned the growing nestlings by placing a video camera on a low tripod below the nest to record the birds entering the nest on the mornings of nestling period days 7–13 inclusive. At least 5 days before video recording started, we caught every bird in the group apart from the dominant female (to avoid the risk of triggering abandonment) to confirm group composition and mark each bird's vent feathers with a uniquely positioned black dye mark to facilitate their identification on provisioning videos. Birds were captured by flushing them from their individual roost chambers at night into a custom capture bag and were returned to their roost chambers once handling was complete. On recording days, video cameras were set up at a standardized time following sunrise to record provisioning for approximately 3 h per nest per day. Within-nest cameras have confirmed that all nest visits in which the birds were not conspicuously carrying grass (events that were readily excluded from our provisioning rate calculations) entail the visiting bird carrying a single food item to the nest and delivering it to the chicks [65]. The videos were subsequently transcribed to determine the timing of each provisioning visit, the duration of the visit (from the bird entering the nest to exiting) and the identity of the provisioning bird (using its unique vent mark and information from its leg rings and sex-specific bill coloration). Whenever the bill of the provisioning bird was visible on their approach to the nest, we also categorized the size of the food item being delivered; simply ‘large’ if it was evidently longer than the bird's bill or ‘small’ if it was not. These data allowed us to conduct analyses (detailed below) of the effects of helper number and developmental rainfall on the overall rates at which broods are provisioned and the probability that provisioned items are large (rather than small).

### (d) Offspring morphological measurements, capture and blood sampling

Our analyses of helper and rainfall effects on offspring mass used body mass measurements (to the nearest 0.01 g; Durascale 100; MyWeigh, Phoenix, AZ) collected routinely from (i) nestlings at day 9 of the nestling provisioning period (the middle of the primary growth phase; nestlings can fledge from day 17), and (ii) fledglings soon after fledging. Fledglings were also assessed for tarsus length and wing length (using calipers, ± 0.1 mm) to complement our measures of body mass with two metrics of body size. We seek fledglings in the field from day 20 and capture any detected fledglings, in the same manner as adult birds (see above), soon afterwards (mean ± s.e. age at fledgling capture in our body mass dataset = 34.38 ± 4.84 days).

Our developmental telomere length estimates stem from blood samples collected from nestlings on day 5 (the soonest after hatching that blood samples could reliably be collected) and day 13 and from fledglings on approximately day 30 (see above). In each case, a small blood sample (< 25 µl) was taken via brachial venepuncture with a 26 G needle for whole blood telomere length determination. Blood was collected in a non-heparinized capillary tube and stored in approximately 500 µl of absolute ethanol at ambient temperature until DNA extraction. DNA was extracted between June and August 2015 (except for two samples extracted in January 2015 and 15 samples extracted in February 2016) and stored in elution buffer at −20°C until quantitative polymerase chain reaction (qPCR) analysis (conducted between August 2015 and September 2016). To control for potential effects of variation in sample storage time prior to DNA extraction, we fitted sample storage duration (from blood sample collection to DNA extraction, accurate to the day) as a covariate predictor in all analyses, following [66].

### (e) DNA extraction and telomere length measurement

Mean telomere length estimates were determined from whole blood samples using qPCR analysis of relative telomere length (referred to as ‘RTL’ here in ‘Methods’ but as ‘telomere length’ for simplicity in ‘Results’ and ‘Discussion’), following [67] with the modifications described below. The following methods have been described previously [37,52,66]. DNA was extracted from whole blood samples using Gentra PureGene Genomic Purification Kits (Qiagen). DNA quantity, quality and integrity were assessed, and any samples that failed were re-extracted or discarded if further extractions also failed [52]. Samples with poor DNA integrity (assessed using gel electrophoresis) were not included in the analyses. DNA concentration and purity were assessed using a NanoVue 4282 spectrophotometer (v.1.7.3) with accepted ratios of between 1.7 and 2.0 for 260/280 and between 1.9 and 2.2 for 260/230. The qPCR method [67] gives a measure of average telomere length per sample relative to a constant copy number control gene, for which we used glyceraldehyde 3-phosphate dehydrogenase (GAPDH) using primers specific to the white-browed sparrow weaver (GAPDH-F 5′AAACCAGCCAAGTATGATGACAT–3′; GAPDH-R 5′–CCATCAGCAGCAGCCTT CA–3′). Telomere primers were as follows: Tel1b 5′–CGGTTTGTTT GGGTTTGGGTTTGGGTTTGGGTTTGGGTT–3′; Tel2b 5′–GGCTTGCCTTACCCTTACC CTTACCCTTACCCTTACCCT–3′.

GAPDH and telomere reactions were carried out on separate 96-well plates on a Stratagene Mx3000 instrument, and each 20 µl reaction contained 5 ng DNA and a master mix containing 10 µl SYBR Green fluorescent dye with low ROX (Brilliant II SYBR qPCR Low ROX Master Mix; Agilent Technologies), with primers at a concentration of 200 nM. Each sample was run in triplicate for both GAPDH and telomere reactions. The thermal cycling schedule for telomere reactions was 15 min at 95°C, followed by 40 cycles of 95°C for 15 s, 57°C for 30 s and 73°C for 30 s. GAPDH reaction thermal cycles were the same, except the annealing temperature was 60°C. Baseline fluorescence was corrected in LinRegPCR [68], and RTL was calculated following [69].

Samples were run in triplicate, as was a between-plate calibration sample (pooled from three birds) and a no-template control, which were included on every plate. A standard curve was created from the 5 ng dilution of the calibration sample for each plate, and efficiencies of GAPDH and telomere reactions were assessed using a 2× serial dilution (from 10 to 0.625 ng) of the calibration sample on every plate. For GAPDH plates, the mean standard curve efficiency was 97.91 (s.d. = 4.05, range = 90.7–109.4) with a mean *r*^2^ of 1.00 (s.d. = 0.003, range = 0.988–1.000). For telomere plates, the mean standard curve efficiency was 98.65 (s.d. = 6.11, range = 85.80–112.80), with a mean *r*^2^ of 1.00 (s.d. = 0.003, range = 0.990–1.000). Mean individual reaction efficiencies were 1.915 for GAPDH reactions (s.d. = 0.082, range = 1.654–2.258) and 1.80 for telomere reactions (s.d. = 0.060, range = 1.560–2.031). Mean reaction *r*^2^ was 0.999 for both GAPDH and telomere reactions (GAPDH and telomere s.d. = 0.001, GAPDH range = 0.999–1.001, telomere range = 0.991–1.000).

All samples collected from a given bird were typically run on the same plate to minimize the impact of plate effects on estimates of within-individual rates of change in telomere length. However, having repeated measures of the same bird nested exclusively within plates (and the possibility of some plates therefore containing only a few birds) could generate challenges teasing bird effects apart from artefactual plate effects in analyses of telomere length. We therefore spread the samples from some birds with many repeat samples (some birds were also sampled later in life for other studies) across plates to avoid having single plates occupied by very few birds. Across all of our telomere length analyses for all studies, we therefore had 258 birds on one plate, 73 birds on two plates, 19 birds on three plates, five birds on four plates and one bird on five plates. Although we attempted to run all samples in triplicate, any replicates with poor amplification (determined by their *C_q_* value) were removed, and the mean RTL for each sample was calculated from the remaining replicates with good amplification. The following equation was used to calculate RTL, where *E*_TEL_ and *E*_GAPDH_ are the mean plate well efficiencies for telomeres and GAPDH, respectively, and the *C_q_* values for the samples reflect the averages across replicates:

$$\begin{array}{r} \text{RTL}=\left(E_{\text{TEL}}\wedge \left(Cq_{\text{TEL}\left[\text{Calibrator}\right]}-Cq_{\text{TEL}\left[\text{Sample}\right]}\right)\right)/\left(E_{\text{GAPDH}}\wedge \left(Cq_{\text{GAPDH}\left[\text{Calibrator}\right]}-Cq_{\text{GAPDH}\left[\text{Sample}\right]}\right)\right). \end{array}$$

We calculated intra-class correlation coefficients (ICCs) to estimate the inter- and intra-plate repeatability of technical replicates using the ‘consistency’ icc function in R (using the irr package [70]). The inter-plate ICC of RTL for samples run on three different plates was 0.567 (two-way model with ‘single units’; 95% confidence interval (CI) = 0.339–0.772, *n* = 23 samples). In order to calculate intra-plate ICC, we converted the *C_q_* values of replicates to an efficiency-corrected starting concentration (*N*_0_) using the PCR plate quantification threshold (*N_q_*) and PCR plate efficiency (*E*) [71]: *N*_0_ = *N_q_* / *E^Cq^*. We estimated the intra-plate ICC of samples using a one-way model with average units. The intra-plate ICC for the efficiency-corrected starting concentration values of the GAPDH reaction triplicates was 0.956 (*n* = 729, 95% CI = 0.950–0.961). For GAPDH reaction duplicates (where a replicate was discarded owing to poor amplification), the ICC was 0.973 (*n* = 36, 95% CI = 0.949–0.986). The intra-plate ICC for the efficiency-corrected starting concentration values of telomere reaction triplicates was 0.947 (*n* = 738, 95% CI = 0.941–0.953) and 0.948 for duplicates (*n* = 27, 95% CI = 0.891–0.976).

### (f) Developmental rainfall

Daily rainfall data were collected from two rainfall gauges located to the west (27°16′58.9″ S, 22°23′02.1″ E) and east (27°17′42.1″ S, 22°27′34.9″ E) of the study site, 7.60 km apart from each other. These two rainfall measurements were highly correlated during the study period (Pearson's product-moment correlation: *r* = 0.875, 95% CI = 0.867–0.882, d.f. = 3347). We therefore calculated average daily values across both gauges for use as a measure of the daily rainfall at the study site. As nestling provisioning and development are likely to be impacted by rain that falls over an extended period (via its effects on environmental food availability), there was a need to identify the biologically relevant time window for our system. For this, we used a rainfall window previously identified as that which best predicts nestling survival from hatching to fledging in our study population [51]: the total rainfall that fell between 36 days prior to hatching and 9 days after hatching, hereafter termed ‘developmental rainfall’.

This ‘developmental rainfall’ window was identified using a sliding window optimization approach (see [51] for detailed methods) considering all possible temporal windows of greater than 4 days in length between an earliest start date of 80 days before the estimated earliest date of fledging for the focal breeding attempt (nestling period day 19) and a latest end date of the estimated earliest date of fledging. Only sliding windows of greater than 4 days in length were considered to decrease the likelihood of false-positive results. For each rainfall window, the total amount of rainfall during that window was calculated for every breeding attempt for which nestling survival to fledging was available (*n* = 400 broods [51]) and then fitted as an additional fixed effect predictor in a ‘baseline model’ of nestling survival for these breeding attempts to test the explanatory power of that rainfall window. The baseline model was a binomial mixed effects model of the proportion of nestlings that survived to fledging, with helper numbers and brood size fitted as fixed effect predictors and breeding season ID (a unique identifier for each of the September-to-April breeding seasons in which data were collected) and mother ID as random effects. The baseline models containing the rainfall predictors stemming from each of the different possible rainfall windows were then ranked by their Akaike information criteria values, and the best-supported rainfall window was chosen (see above). The comparisons of the alternative rainfall windows yielded clear evidence of a single ‘hotspot’ of explanatory power centred on this window, and 10 randomizations confirmed that the explanatory power of this rainfall window was far higher than that which would be expected by chance if rainfall and nestling survival were actually unrelated [51].

### (g) Statistical methods

All statistical methods were carried out in ‘R’ (v.4.4.2; [72]). We fitted the full models described below and only removed initially fitted interactive terms if they were not significant. Otherwise, no model simplification or selection occurred. Fixed-effect statistical significance was calculated using likelihood-ratio tests comparing the explanatory power of the full model with and without the focal fixed effect. Model assumptions (e.g. Gaussian distribution of residuals and overdispersion) were checked for using the R packages DHARMa (v.0.4.7 [73]) and performance (v.0.15.3 [74]). We found no evidence for deviation from model assumptions in any case.

#### (i) Effects of helpers and developmental rainfall on brood provisioning

Variation in the number of feeds received by a brood was modelled using generalized linear mixed models (LMMs) (GLMMs) with a Poisson error distribution. These Poisson models included the duration of the observation period (the number of hours for which the focal nest was observed on the focal day) as an offset and thus effectively modelled provisioning rate. Variation in the proportion of these provisioning visits in which the delivered food item was large (as opposed to small) was also modelled using a GLMM, this time with binomial error distribution (1 for large and 0 for small). Both sets of provisioning models included the same fixed and random effects as predictors. Developmental rainfall (see above; mean-centred and divided by one standard deviation; i.e. ‘scaled’), helper number (calculated as the number of subordinate group members older than six months between days 1 and 24 of the breeding attempt; helper numbers typically remain constant throughout this time), brood size (calculated as the number of nestlings on the day that the provisioning observations were taken) and brood age (in days since the hatching of the first chick in the clutch) were included as fixed effects along with the interaction between developmental rainfall and helper number. Four terms were included as random intercepts: season ID (a unique identifier for each of the September-to-April breeding seasons in which data were collected), social group ID (the social group in which the focal clutch was being reared), clutch ID (the focal clutch being reared) and date ID (that date of provisioning data collection). Date ID captured the date on which provisioning was measured and was fitted as a random effect only in this model because provisioning rates (unlike the physiological metrics analysed later) can change radically among days owing to variation, for example, in weather conditions and predation risk. Each Julian date was fitted as a separate level of the date ID factor and could have repeated measures, as provisioning rate was often monitored across multiple groups on the same day. The provisioning datasets comprised 342 daily measures of provisioning rate (and proportion of food items delivered of determinable size class that were large) collected across nine seasons from 35 social groups rearing 138 broods.

A common concern in studies of the effects of helper numbers on performance in cooperative breeders is that positive correlations between the two could arise not from a causal effect of helpers but instead from both helper numbers and the performance trait being positively impacted by transient resource peaks or variation in parental and/or territory quality [19,25,75]. To address the possibility that transient resource peaks could increase both helper number (by yielding additional young helpers) and current productivity, leaving the two positively correlated, we excluded young individuals (< 6 months old) from our calculations of helper numbers (they also contribute little to helping [55]). To then address the possibility that variation in helper number is being confounded by variation in parental and/or territory quality, we took the following approach. Having detected a significant positive relationship between helper number and provisioning rate in the analysis above, we ran a second model with the same structure but having partitioned the helper number predictor into two separate predictors capturing its within- and among-mother components (following [75,76]): *Δ* helper number and *µ* helper number, respectively. *µ* helper number is the mean helper number that a mother had across all of her breeding attempts in the relevant dataset, while *Δ* helper number is the difference between her helper number in the focal brood and *µ* helper number. Here we focused our attention on mothers, as mothers provision offspring at substantially higher rates than fathers [77]. This partitioning approach allowed us to achieve two things. First, we verified that there was a significant positive effect of *within-mother* variation in helper number on provisioning rate (and hence within-territory variation, as each mother only bred on one territory), indicating that the effect of helper number on provisioning rate detected in the initial analysis cannot be attributed solely to confounding variation in quality among mothers or their territories [75,76]. Second, we established that the effect of helper number on provisioning rate detected in the initial analysis is not contributed to by appreciable confounding variation in provisioning rates among mothers or their territories (because the effect size for among-mother variation in helper number matched that for within-mother variation in helper number [75,76], and therefore, both matched the overall effect size for helper number prior to partitioning).

#### (ii) Effects of helpers and developmental rainfall on offspring growth

Variation in the body masses of nestlings on day 9 of the breeding attempt (day 1 denoting the day on which the first chick hatched; see above) was analysed using an LMM (i.e. assuming a Gaussian distribution of residuals). Developmental rainfall, helper number, nestling age (days since the focal nestling was first seen to have hatched; we make daily afternoon nest checks of hatching clutches; see above), brood size and the interaction between developmental rainfall and helper number were included as fixed effects. Four terms were included as random intercepts: season ID, social group ID, mother ID and clutch ID. Variation in the body masses of fledglings (between 30 and 45 days of age) was also analysed using an LMM, including the same fixed and random effects as the model for nestling mass, but with the addition of sex (male, female or unknown) as a three-level factor fixed effect. We did initially include nestling sex as an additional fixed effect predictor in the model of nestling body mass at breeding attempt day 9 as well. However, this revealed no sex difference in body mass (i.e. between males and females) at that age but did capture an effect in which nestlings of unknown sex were lighter than nestlings of known sex. As this difference probably reflects an artefactual reverse causal relationship, in which a nestling being lighter on day 9 causes it to ultimately be of unknown sex (by reducing its chance of survival to blood sampling for molecular sexing; lighter nestlings being less likely to survive in this species [37]), we removed nestling sex from the predictor set under consideration at that age, having confirmed that doing so did not have a qualitative impact on the outcomes of the model. For example, when including sex as a predictor, the detected interaction between helper number and rainfall (the primary result of interest) was, if anything, more significant ($\chi_{1}^{2}$ = 5.13, *p* = 0.024) than in the model that we present without sex as a predictor ($\chi_{1}^{2}$ = 4.02, *p* = 0.045). Our analysis of body mass at breeding attempt day 9 used a dataset of body masses for 435 nestlings within 279 broods hatched to 62 mothers in 35 groups. However, our analysis of fledgling body mass used a dataset of body masses for 133 fledglings from 103 broods hatched to 47 mothers in 34 groups.

Having completed the above analyses, we re-ran the models of body mass at both ages, including the mean egg volume for the clutch (as calculated in [53]) as an additional fixed effect predictor, to control for potential confounding effects of variation in maternal prenatal investment in the egg (which could itself covary with helper numbers and rainfall [53]). We used the mean egg volume for the clutch as it was often not possible to link individual nestlings back to the egg from which they hatched, and because variation among clutches in mean egg volume accounts for 75% of the overall variation in egg volume in this species (based on the analysis of 490 eggs laid across 271 clutches over the course of our long-term field study [53]). This approach reduced the sample sizes available for the two analyses to masses for 408 offspring from 261 clutches at day 9 and 122 offspring from 94 clutches at fledging.

#### (iii) Effects of helpers and developmental rainfall on developmental telomere attrition

We used an LMM to investigate the predictors of variation in the telomere length of nestlings during the developmental period (range = 4–35 days of age, mean = 14 days of age). In order to model the within-individual rate of change in telomere length, we partitioned variation in offspring age into two predictors (following [75,76]) capturing the within-individual change in age (‘offspring *Δ* age’) and the among-individual variation in mean age at sampling (‘offspring *µ* age’). Offspring *µ* age is the mean age at which the focal offspring was sampled across all of its RTL measures in this dataset, while offspring *Δ* age is the difference between their age at which the focal RTL measure was taken and their offspring *µ* age. This partitioning approach allowed us to model the effects of helper number and developmental rainfall on the within-individual rate of change in RTL by fitting as predictors the interactions between both variables and offspring *Δ* age. We also fitted the following fixed effects as predictors: offspring *µ* age, sample storage time (the time elapsed in days between blood sample collection and DNA extraction), brood size at hatching and offspring sex. Six terms were included as random intercepts: season ID, mother ID, social group ID, clutch ID, nestling ID and PCR plate ID (to account for among-plate variation in estimates of RTL). This analysis used a dataset comprising 168 RTL measures collected from 74 offspring hatched from 56 clutches laid by 34 mothers in 32 social groups. All offspring included in the dataset had either two or three RTL measures available, collected at different ages within the first 36 days of life, allowing them to contribute to the estimation of a slope for offspring *Δ* age. Offspring with only a single measure of RTL available were not included, as these offspring would be uninformative with regard to the within-individual rate of change in RTL over time (i.e. offspring *Δ* age) and hence the impacts of helper number and developmental rainfall on this. Estimates of the percentage of variance explained by different predictors were obtained through 100 bootstrap iterations using the R package partR2 [78].

As the above analysis of offspring telomere lengths revealed an interaction between helper number and offspring *Δ* age (indicating that helper number predicted the within-individual rate of change in RTL during development), we conducted three follow-up analyses. First, to investigate whether this relationship could be a product in part of a pre-existing relationship between helper number and hatchling RTL arising from the prenatal developmental period, we investigated the effects of helper number and developmental rainfall on the RTL of nestlings soon after hatching (a single RTL estimate per nestling taken at 4–7 days of age, mean age = 5.44 days; it was not possible to reliably obtain blood samples from nestlings prior to this age). The same covariates and random effects were fitted as described for the telomere length model above, except offspring age was fitted as an unpartitioned variable and nestling ID was not fitted as a random effect (given the lack of repeated measures per nestling in this analysis). The dataset comprised single RTL measures from 84 hatchlings from 58 clutches raised by 39 mothers in 30 groups. Second, to investigate whether this relationship could be a product in part of changes in maternal egg investment, we repeated this analysis while also fitting the mean egg volume of the clutch as a predictor (doing so did not impact the sample size). Third, we investigated the effects of helper number and developmental rainfall on the RTL of fledglings (a single RTL estimate per fledgling, taken at 30–36 days of age; mean age = 30.90 days). This analysis contained the same covariate predictors and random intercept terms as the model of RTL soon after hatching and used a dataset of single RTL measures from 50 fledglings from 45 clutches raised by 27 mothers in 26 groups.

## 3. Results

### (a) The effects of helpers and developmental rainfall on brood provisioning

The overall rate at which broods were fed was positively predicted by both helper number (effect size following back-transformation into the data scale (our tables report effect sizes in the link scale) ± s.e. = + 1.07 ± 0.05 feeds h^−1^ per additional helper; $\chi_{1}^{2}$ = 10.91, *p* = 0.001; table 1; figure 1a) and developmental rainfall (effect size in the data scale ± s.e. = + 1.08 ± 0.04 feeds h^−1^ per standard deviation in rainfall; $\chi_{1}^{2}$ = 4.16, *p* = 0.041; table 1; figure 1b). There was no significant interaction between helper numbers and developmental rainfall ($\chi_{1}^{2}$ = 0.09, *p* = 0.759). Provisioning rates were also positively predicted by brood size ($\chi_{1}^{2}$ = 28.05, *p* < 0.001; table 1) and brood age ($\chi_{1}^{2}$ = 12.07, *p* = 0.001; table 1). Developmental rainfall also positively predicted the probability that a provisioning visit involved the delivery of a large food item rather than a smaller one ($\chi_{1}^{2}$ = 5.21, *p* = 0.022; table 2). Provisioned food item size was unrelated to helper number, brood size and brood age (all $\chi_{1}^{2}$ < 2.00, *p* > 0.150; table 2) and was not significantly predicted by the interaction between helper number and developmental rainfall ($\chi_{1}^{2}$ = 0.49, *p* = 0.480).

**Table 1.** Factors affecting the overall rate at which broods are provisioned. (Model estimates, standard errors (s.e.) and their 95% confidence intervals (95% CI) are provided along with results from likelihood-ratio tests (*χ*^2^) assessing the statistical significance of each predictor within the full model (i.e. a model containing all terms in the table above). *n* = 342 daily measures of provisioning rate collected across nine seasons from 35 social groups rearing 138 broods. Random effect standard deviations (in log feeds h^−1^): ‘clutch ID’ = 0.36, ‘date’ = 0.31, ‘group ID’ = 0.00 and ‘season’ = 0.00. The interaction between helper number and developmental rainfall did not significantly explain variation in provisioning rates ($\chi_{1}^{2}$ = 0.09, *p* = 0.759). Developmental rainfall was mean-centred and scaled by one standard deviation prior to model fitting to improve model convergence. Model estimates are presented in the link scale (log). d.f. = degrees of freedom.

fixed effect	estimate	s.e.	95% CI	*χ*^2^	d.f.	*p*-value
intercept	1.10	0.22	0.68, 1.53			
developmental rainfall (mm)	0.08	0.04	0.00, 0.16	4.16	1	**0.041**
helper number	0.07	0.02	0.03, 0.11	10.91	1	**0.001**
brood age	0.05	0.02	0.02, 0.08	12.07	1	**0.001**
brood size	0.38	0.07	0.25, 0.51	28.05	1	**<0.001**

The *p*-values of statistically significant terms (*p* < 0.05) are highlighted in bold.

**Figure 1.**
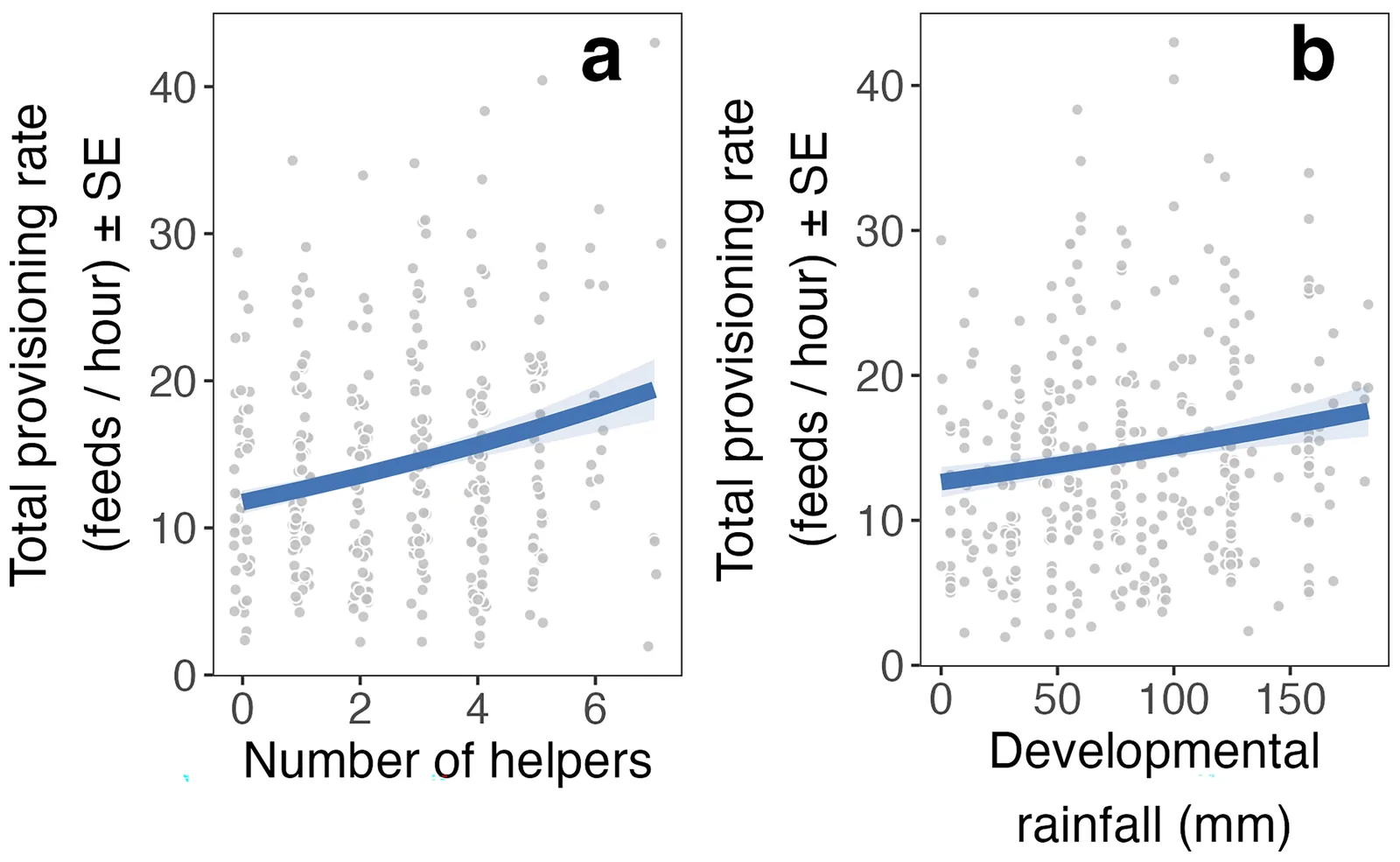
Effects of helper number and developmental rainfall on the overall rate at which broods are provisioned. Total provisioning rate to the brood (feeds h^−1^) was positively predicted by (a) the number of helpers attending the brood and (b) the amount of rainfall that fell between 36 days before hatching and 9 days after hatching (‘developmental rainfall’; see ‘Methods’). Grey points represent raw data, whereas thick blue lines and shaded areas give mean model predictions ± standard error (s.e.) (table 1).

**Table 2.** Factors affecting the probability that a provisioned food item is large (rather than small). (Model estimates, standard errors (s.e.) and their 95% confidence intervals (95% CI) are provided along with results from likelihood-ratio tests (*χ*^2^) assessing the statistical significance of each predictor within the full model (i.e. a model containing all terms in the table above). Sample sizes are the same as in table 1. Random effect standard deviations (in logit feeds h^−1^): ‘clutch ID’ = 1.23, ‘date’ = 0.73, ‘group ID’ = 0.35 and ‘season’ = 1.25. The interaction between helper number and developmental rainfall did not significantly explain variation in the proportion of provisioned prey items that were large ($\chi_{1}^{2}$ = 0.49, *p* = 0.483). Developmental rainfall was mean-centred and scaled by one standard deviation prior to model fitting, to improve model convergence. Model estimates are presented in the link scale (logit). d.f. = degrees of freedom.

fixed effect	estimate	s.e.	95% CI	*χ* ^2^	d.f.	*p*-value
intercept	−2.64	0.93	−4.47, −0.82			
developmental rainfall (mm)	0.37	0.16	0.06, 0.68	5.21	1	**0.022**
helper number	−0.02	0.09	−0.20, 0.16	0.03	1	0.853
brood age	−0.08	0.06	−0.20, 0.03	1.90	1	0.168
brood size	−0.32	0.27	−0.84, 0.20	1.33	1	0.249

The *p*-values of statistically significant terms (*p* < 0.05) are highlighted in bold.

Partitioning variation in helper numbers into its within- and among-mother (and hence within- and among-territory) components revealed that within-mother variation in helper numbers positively predicted overall provisioning rate (*Δ* helper number effect size in the data scale ± s.e. = +1.07 ± 0.04 feeds h^−1^ per additional helper; $\chi_{1}^{2}$ = 6.95, *p* = 0.008; table 3), confirming that the overall association with helper numbers cannot be attributed solely to confounding variation in quality among mothers or their territories. Moreover, the effect size for within-mother variation in helper number was very similar to that for among-mother variation in helper number (*µ* helper number effect size in the data scale ± s.e. = +1.08 ± 0.05 feeds h^−1^ per additional helper; $\chi_{1}^{2}$ = 5.86, *p* = 0.016; table 3) and, therefore, the overall effect of helper number prior to partitioning (see above; table 1). This alignment of effect sizes within and among mothers suggests that the overall population-level relationship between helper number and provisioning rate arises principally from the within-mother effect of helper number (which will cause a comparable among-mother effect in these circumstances), without appreciable additional inputs from confounding variation among mothers or their territories [75,76].

**Table 3.** Factors affecting the overall rate at which broods are provisioned, after partitioning the effect of helper numbers into its within- and among-mother components. (Model estimates, standard errors (s.e.) and their 95% confidence intervals (95% CI) are provided along with results from likelihood-ratio tests (*χ*^2^) assessing the statistical significance of each predictor within the full model (i.e. a model containing all terms in the table above). Sample sizes are the same as in table 1. Random effect standard deviations (in log feeds h^−1^): ‘clutch ID’ = 0.36, ‘date’ = 0.31, ‘group ID’ = 0.00 and ‘season’ = 0.00. Developmental rainfall was mean-centred and scaled by one standard deviation prior to model fitting to improve model convergence. Model estimates are presented in the link scale (log). d.f. = degrees of freedom.

fixed effect	estimate	s.e.	95% CI	*χ*^2^	d.f.	*p*-value
intercept	1.08	0.23	0.41, 1.44			
developmental rainfall (mm)	0.08	0.04	0.00, 0.16	4.15	1	**0.042**
*Δ* helper number	0.07	0.03	0.02, 0.12	6.95	1	**0.008**
*µ* helper number	0.08	0.03	0.02, 0.14	5.86	1	**0.016**
brood age	0.05	0.02	0.02, 0.09	12.24	1	**<0.001**
brood size	0.38	0.07	0.25, 0.51	28.19	1	**<0.001**

The *p*-values of statistically significant terms (*p* < 0.05) are highlighted in bold.

### (b) The effects of helpers and developmental rainfall on offspring growth

The mass of individual nestlings at 7–9 days of age was predicted by a significant interaction between helper number and developmental rainfall (figure 2; $\chi_{1}^{2}$ = 4.02, *p* = 0.045; table 4), in which helpers had more strongly positive effects during periods of lower developmental rainfall (i.e. during harsher climatic conditions in this semi-arid region; figure 2). Nestling mass at 7–9 days of age was also positively predicted by nestling age ($\chi_{1}^{2}$ = 82.03, *p* < 0.001; table 4) but was unrelated to brood size ($\chi_{1}^{2}$ = 1.42, *p* = 0.233; table 4). This interactive effect of helper number and developmental rainfall on nestling mass was still significant ($\chi_{1}^{2}$ = 4.88, *p* = 0.027; supplementary material, table S1) if we included in the model as an additional fixed effect predictor the mean egg volume for the focal clutch (which strongly predicts hatchling mass [53] and, in this model, also positively predicted nestling mass at 7–9 days of age; $\chi_{1}^{2}$ = 14.32, *p* < 0.001; supplementary material, table S1). The detected interaction therefore probably reflects effects of helper numbers and developmental rainfall on the postnatal growth of nestlings, rather than correlated effects of maternal plasticity in egg investment on prenatal growth.

**Table 4.** Factors affecting offspring mass at 7–9 days of age. (Model estimates, standard errors (s.e.) and their 95% confidence intervals (95% CI) are provided along with results from likelihood-ratio tests (*χ*^2^) assessing the statistical significance of each predictor within the full model (i.e. a model containing all terms in the table above). *n* = 435 nestling mass measurements were collected across nine seasons from 35 social groups rearing 279 clutches. Random effect standard deviations (in g): ‘clutch ID’ = 2.39, ‘group ID’ = 0.00, ‘mother ID’ = 8.78 and ‘season’ = 1.50. Developmental rainfall was mean-centred and scaled by one standard deviation prior to model fitting to improve model convergence. d.f. = degrees of freedom.

fixed effect	estimate	s.e.	95% CI	*χ*^2^	d.f.	*p*-value
intercept	18.65	1.17	16.37, 20.94			
developmental rainfall (mm)	1.86	0.40	1.08, 2.65			
helper number	0.18	0.15	−0.11, 0.47			
nestling age (days)				82.03	2	**<0.001**
7	–	–	–			
8	2.61	0.60	1.43, 3.78			
9	5.72	0.63	4.48, 6.96			
brood size	−0.51	0.42	−1.32, 0.31	1.42	1	0.233
developmental rainfall (mm) × helper number	−0.23	0.12	−0.46, 0.00	4.02	1	**0.045**

The *p*-values of statistically significant terms (*p* < 0.05) are highlighted in bold.

**Figure 2.**
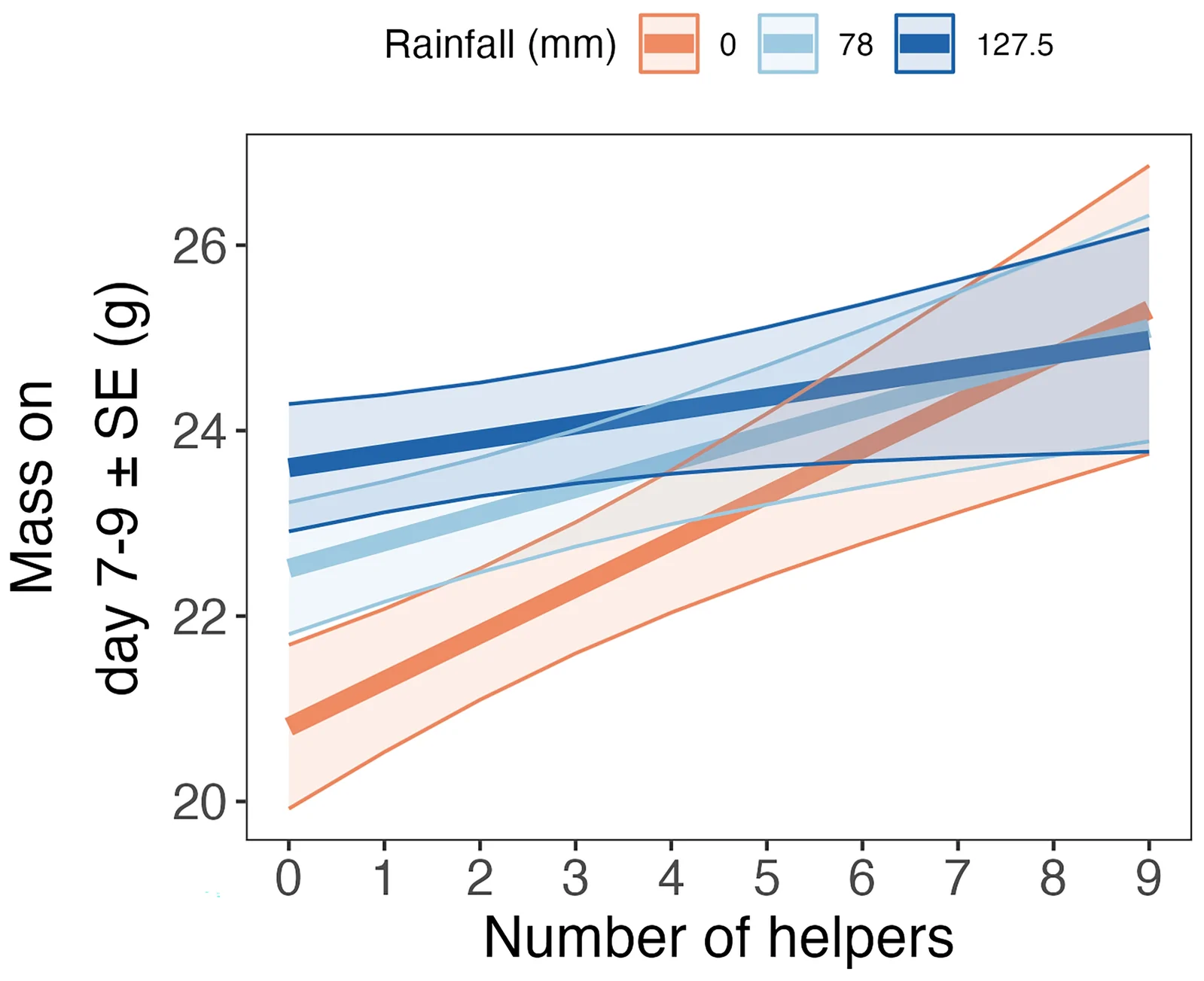
Effects of helper number and developmental rainfall on offspring mass at 7–9 days of age. Helper numbers had a positive effect on nestling mass at 7–9 days of age, the strength of which reduced with increasing rainfall (e.g. moving from the orange to the light blue to the dark blue lines; helper number × developmental rainfall interaction: $\chi_{1}^{2}$ = 4.02, *p* = 0.045; table 4). Coloured lines and shared areas represent model predictions (± standard error (s.e.)) for developmental rainfall and helper number effects on nestling mass. Model predictions are drawn for no rainfall (orange) and the first and third tertiles of the non-zero rainfall distribution (78 and 127.5 mm, light and dark blue, respectively). This was done for illustration purposes; developmental rainfall was included as a continuous variable in the model.

The mass of individual fledglings at 30–45 days of age (i.e. after fledging from the nest) was significantly positively predicted by developmental rainfall ($\chi_{1}^{2}$ = 3.94, *p* = 0.047; table 5) and their sex (males were on average (± s.e.) 2.99 ± 0.55 g heavier than females; *χ*^2^ = 28.79, *p* < 0.001; table 5). Fledgling mass was not significantly predicted by helper number, brood size or fledgling age at measurement (*χ*^2^ < 0.75, *p* > 0.39 for all; table 5). This significant positive effect of rainfall on fledgling mass appears to be a product of an overall larger body size among fledglings reared in wetter conditions, as fledgling tarsus length was also significantly positively predicted by developmental rainfall (effect size ± s.e. = +0.14 ± 0.07 mm per standard deviation of rainfall; $\chi_{1}^{2}$ = 4.21, *p* = 0.040; supplementary material, table S2), and fledgling wing length also showed a tendency towards a positive relationship with developmental rainfall (effect size ± s.e. = +0.70 ± 0.43 mm per standard deviation of rainfall; *χ*^2^ = 2.95, *p* = 0.086; supplementary material, table S3). Neither fledgling tarsus length nor wing length was predicted by helper numbers (*χ*^2^ < 0.16 and *p* > 0.70 for both; supplementary material, tables S2 and S3).

**Table 5.** Factors affecting fledgling mass (between 30 and 45 days of age). (Model estimates, standard errors (s.e.) and their 95% confidence intervals (95% CI) are provided along with results from likelihood-ratio tests (*χ*^2^) assessing the statistical significance of each predictor within the full model (i.e. a model containing all terms in the table above). *n* = 133 fledgling mass measurements were collected across nine seasons from 34 social groups rearing 103 clutches. Random effect standard deviations (in g): ‘clutch ID’ = 0.00, ‘group ID’ = 0.92, ‘mother ID’ = 0.81 and ‘season’ = 1.86. Developmental rainfall was mean-centred and scaled by one standard deviation prior to model fitting to improve model convergence. The interaction between helper number and developmental rainfall did not significantly explain variation in fledgling mass ($\chi_{1}^{2}$ = 0.58, *p* = 0.448). d.f. = degrees of freedom.

fixed effect	estimate	s.e.	95% CI	*χ*^2^	d.f.	*p*-value
intercept	39.81	2.70	34.51, 45.10			
developmental rainfall (mm)	0.73	0.37	0.01, 1.45	3.94	1	**0.047**
helper number	−0.09	0.24	−0.56, 0.39	0.06	1	0.802
age (days)	−0.02	0.07	−0.15, 0.10	0.08	1	0.783
brood size	−0.44	0.51	−1.44, 0.56	0.72	1	0.395
offspring sex				28.79	2	**<0.001**
female	–	–	–			
male	2.99	0.55	1.92, 4.07			
unknown	3.10	1.29	0.58, 5.63			

The *p*-values of statistically significant terms (*p* < 0.05) are highlighted in bold.

### (c) The effects of helpers and rainfall on developmental telomere attrition

The rate of change in mean whole blood telomere length during the postnatal-dependent period (4–45 days of age) was modelled as the effect of within-individual change in age (‘offspring *Δ* age’ in table 6) on the telomere lengths of offspring. Our modelling revealed a significant interaction between helper numbers and the within-individual change in age (helper number × offspring *Δ* age; *χ*^2^ = 8.12, *p* = 0.004; table 6), indicating that the within-individual rate of change in telomere length with age depends upon helper number. Offspring that were reared by more helpers showed reduced developmental telomere attrition rates (figure 3). In the absence of helpers, developing nestlings experienced significant reductions in mean telomere length with advancing age: effect of offspring *Δ* age ± s.e. = −0.006 ± 0.002, *p* = 0.006 (*n* = 35 samples from 17 offspring in 12 clutches born to 10 mothers; re-running the table 6 model for data with helper number = 0). In the presence of helpers, developing nestlings did not experience significant changes in telomere length with advancing age: effect of offspring *Δ* age ± s.e. = +0.002 ± 0.001, *p* = 0.224 (*n* = 133 samples from 57 offspring in 44 clutches born to 30 mothers; re-running the table 6 model for data with helper number >0).

**Table 6.** Factors affecting offspring mean whole blood telomere length across the primary growth period (4–35 days of age). (Model estimates, standard errors (s.e.) and their 95% confidence intervals (95% CI) are provided along with results from likelihood-ratio tests (*χ*^2^) assessing the statistical significance of each predictor within the full model (i.e. a model containing all terms in the table above). *n* = 168 telomere length estimates were collected across five seasons from 74 offspring in 32 social groups rearing 56 clutches. Random effect standard deviations: ‘clutch ID’ = 0.09, ‘group ID’ = 0.00, ‘individual ID’ = 0.05, ‘mother ID’ = 0.03, ‘season’ = 0.00 and ‘PCR plate ID’ = 0.09. Developmental rainfall and storage time were mean-centred and scaled by one standard deviation prior to model fitting to improve model convergence. The following interactions did not explain significant variation in telomere length and so were removed from the model: *Δ* age x developmental rainfall ($\chi_{1}^{2}$ = 1.42, *p* = 0.233), *Δ* age × mean age ($\chi_{1}^{2}$ = 1.52, *p* = 0.216) and helper number × mean age ($\chi_{1}^{2}$ = 1.38, *p* = 0.241). d.f. = degrees of freedom.

fixed effect	estimate	s.e.	95% CI	*χ*^2^	d.f.	*p*-value
intercept	0.9914	0.1074	0.7810, 1.2018			
developmental rainfall (mm)	0.0226	0.0261	−0.0286, 0.0738	0.78	1	0.378
helper number	−0.0120	0.0165	−0.0445, 0.0204			
*Δ* age (days)	−0.0038	0.0018	−0.0073, −0.0004			
*µ* age (days)	0.0056	0.0046	−0.0035, 0.0146	1.52	1	0.217
storage (days)	−0.0305	0.0252	−0.0799, 0.0190	1.59	1	0.207
brood size	−0.0237	0.0344	−0.0911, 0.0436	0.51	1	0.476
offspring sex				0.52	1	0.471
female	–	–	–			
male	0.0293	0.0368	−0.0427, 0.1013			
helper number × *Δ* age (days)	0.0019	0.0006	0.0006, 0.0031	8.12	1	**0.004**

The *p*-values of statistically significant terms (*p* < 0.05) are highlighted in bold.

**Figure 3.**
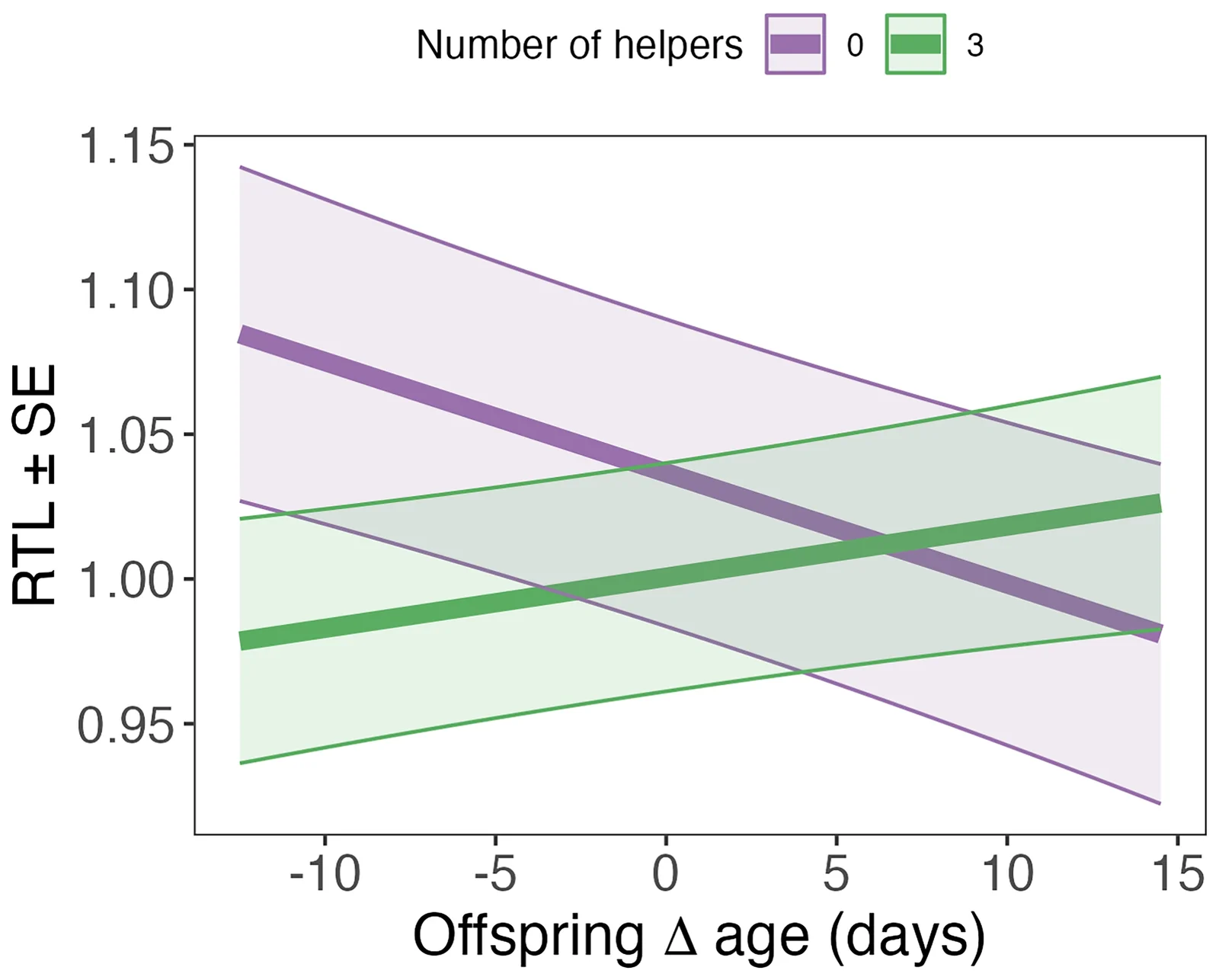
The effect of helper numbers on the within-individual change in telomere length with advancing offspring age (range: 4–35 days of age). Offspring *Δ* age reflects within-individual change in age, relative to the mean age of sampling for the focal individual (see ‘Methods’). Offspring that were reared by more helpers experienced less severe within-individual age-related declines in mean whole blood telomere length (i.e. lower telomere attrition rates, reflected as an offspring *Δ* age × helper number interaction: $\chi_{1}^{2}$ = 8.12, *p* = 0.004; table 6). Coloured lines and shaded areas represent model predictions (± standard error (s.e.)), drawn for offspring reared by the breeding pair either with no helpers (purple; 35 observations) or the median helper number when helpers were present (three helpers; green; 133 observations). This categorization was done for illustration purposes; the helper number was included as a continuous variable in the model.

Developmental rainfall did not predict telomere length, either when fitted as a main effect (*χ*^2^ = 0.78, *p* = 0.378; table 6) or in an interaction with the within-individual change in age (*χ*^2^ = 1.39, *p* = 0.239; table 6; which models the effect of rainfall on the rate of change in telomere length). Telomere length was not significantly predicted by brood size, offspring sex or sample storage time prior to extraction (*χ*^2^ < 1.60, *p* > 0.20 for all; table 6).

The relationship between helper number and the rate of change in telomere length during the postnatal-dependent period is unlikely to be a by-product of correlated variation in maternal prenatal investment impacting telomere length at hatching, as the mean whole blood telomere lengths of nestlings soon after hatching (less than 7 days of age) were not significantly predicted by helper number or developmental rainfall (*χ*^2^ < 0.95, *p* > 0.33 for both; table 7) or the interaction between the two (*χ*^2^ = 1.423, *p* = 0.233). Fitting the mean egg volume for the clutch as an additional predictor in this model also yielded no evidence that variation in maternal investment in the egg impacted telomere length soon after hatching (*χ*^2^ = 2.70, *p* = 0.101; supplementary material, table S4). The helper number also doesn't predict telomere length when re-running the table 7 model with only the first available sample per nestling from the table 6 model (i.e. the measure with the lowest *Δ* age value for each nestling): helper number effect ± s.e. = −0.021 ± 0.020, *χ*^2^ = 1.498, *p* = 0.221.

**Table 7.** Factors affecting nestling mean whole blood telomere length soon after hatching (less than 7 days of age). (Model estimates, standard errors (s.e.) and their 95% confidence intervals (95% CI) are provided along with results from likelihood-ratio tests (*χ*^2^) assessing the statistical significance of each predictor within the full model (i.e. a model containing all terms in the table above). *n* = 84 telomere length estimates from 84 nestlings were collected across five seasons from 30 social groups rearing 58 clutches. Random effect standard deviations: ‘clutch ID’ = 0.11, ‘group ID’ = 0.00, ‘mother ID’ = 0.00, ‘season’ = 0.10 and ‘PCR plate ID’ = 0.08. Developmental rainfall and storage time were mean-centred and scaled by one standard deviation prior to model fitting to improve model convergence. d.f. = degrees of freedom)

fixed effect	estimate	s.e.	95% CI	*χ*^2^	d.f.	*p*-value
intercept	1.27	0.27	0.75, 1.80			
developmental rainfall (mm)	0.04	0.04	−0.04, 0.11	0.93	1	0.334
helper number	0.00	0.02	−0.03, 0.04	0.15	1	0.702
nestling age (days)	−0.05	0.04	−0.13, 0.03	1.30	1	0.254
storage (days)	0.03	0.07	−0.10, 0.17	0.22	1	0.639
brood size	0.02	0.04	−0.07, 0.10	0.11	1	0.742
sex				0.27	1	0.602
female	–	–	–			
male	0.01	0.05	−0.08, 0.11			

The relationship between helper number and the within-individual rate of change in telomere length during the dependent period (4–45 days of age; table 6) did not give rise to a significant positive effect of helper number on the mean telomere lengths of fledglings (table 8). Telomere length at fledging was not significantly predicted by helper number, developmental rainfall, brood size, fledgling age, fledgling sex or sample storage time (*χ*^2^ <1.50, *p* > 0.20 for all; table 8). That the relationship between helper number and the rate of change in telomere length among nestlings (i.e. the helper number × *Δ* age interaction in table 6) did not give rise to a significant effect of helper numbers on absolute telomere length at fledgling may simply reflect this interaction being a modest source of variation in nestling telomere length estimates (e.g. explaining 1.6% of variation in the table 6 model) relative to other sources, such as inter-individual variation (e.g. individual ID explains 6.1%, clutch ID 22.84% and group ID 15.37%) and inter-plate variation (e.g. PCR plate ID explains 19.4%), which could themselves arise from a range of mechanisms unrelated to somatic integrity (see ‘Discussion’).

**Table 8.** Factors affecting fledgling mean whole blood telomere length (30–36 days after hatching). (Model estimates, standard errors (s.e.) and their 95% confidence intervals (95% CI) are provided along with results from likelihood-ratio tests (*χ*^2^) assessing the statistical significance of each predictor within the full model (i.e. a model containing all terms in the table above). *n* = 50 telomere length estimates were collected from 50 fledglings across 6 seasons from 26 social groups rearing 45 clutches. Random effect standard deviations: ‘clutch ID’ = 0.15, ‘group ID’ = 0.00, ‘mother ID’ = 0.12, ‘season’ = 0.07 and ‘PCR plate ID’ = 0.00. Developmental rainfall and storage time were mean-centred and scaled by one standard deviation prior to model fitting to improve model convergence. d.f. = degrees of freedom.)

fixed effect	estimate	s.e.	95% CI	*χ*^2^	d.f.	*p*-value
intercept	1.95	0.84	0.30, 3.60			
developmental rainfall (mm)	0.01	0.04	−0.07, 0.09	0.62	1	0.430
helper number	0.00	0.03	−0.05, 0.06	0.25	1	0.617
fledgling age (days)	−0.03	0.03	−0.08, 0.02	1.49	1	0.222
storage (days)	−0.01	0.04	−0.10, 0.07	0.44	1	0.506
brood size	−0.06	0.06	−0.17, 0.06	1.08	1	0.298
sex				0.08	1	0.783
female	–	–	–			
male	0.01	0.07	−0.12, 0.15			

## 4. Discussion

We investigated the effect of helping behaviour on offspring growth and telomere dynamics in a wild cooperative bird, the white-browed sparrow weaver. Our analyses first confirmed that helper numbers and recent rainfall are key predictors of offspring provisioning rate. Accordingly, helpers appear to have beneficial effects on the growth and telomere dynamics of offspring. Helper numbers positively predicted growth (reflected by offspring mass at eight days of age) but did so more strongly in drier conditions (reflected by an interaction between helper number and rainfall in which helpers reduced rainfall-induced variance in growth). This finding suggests that helpers buffer offspring growth against variation in rainfall in this unpredictable rainfall regime and is notable in the context of the global association between avian cooperative breeding and variable rainfall regimes [51,79–82] (see below). Our analyses of telomere dynamics also suggest beneficial effects of helping, as the within-individual rate of change in telomere length in offspring was positively predicted by helper numbers (reflected by an interaction between helper number and offspring *Δ* age). As predicted, offspring fed by more helpers showed lower telomere attrition rates during the helping period, the first result of its kind for cooperative breeders. Specifically, nestlings reared in the absence of helpers (i.e. by unaided parents) experienced significant reductions in mean telomere length with advancing age, while nestlings reared with the aid of helpers did not. Here, we consider the extent to which these relationships between helper numbers and offspring provisioning, growth and telomere dynamics reflect causal effects of helping, the mechanisms by which they arise, and their implications for our understanding of helper effects in cooperative societies and the global distribution of avian cooperative breeding [79–82].

### (a) The causality of relationships with helper number

While relationships between helper numbers and recipient performance could be causal (as hypothesized here), a commonly plausible alternative explanation is that they instead reflect effects of correlated variation in territory and/or parental quality, as helpers (commonly being surviving offspring from previous broods on the territory) are likely to recruit in higher numbers to higher quality territories or parents [19,25,75]. However, our analyses of provisioning rates (the primary mechanism by which helper numbers and territory and parental quality are likely to impact postnatal growth and telomere dynamics) suggest that this is unlikely to be the case here. Partitioning of the statistical effect of helper number on provisioning rate into its within- and among-mother components (following [75,76], and as mothers provision offspring at substantially higher rates than fathers in this species [77]) revealed two key findings. First, there is a significant positive effect of within-mother variation in helper number, indicating a positive relationship between helper numbers and provisioning rate that cannot be attributed to variation *among* mothers (or their territories, as each mother only breeds on one territory). Second, the magnitude of the among-mother effect size matches that of the within-mother effect size (and thus both align with the overall effect size for helper number prior to partitioning). This suggests that the overall relationship between helper number and provisioning rate is not appreciably confounded by correlated variation in provisioning rates *among* mothers or their territories [75,76]. Specifically, mothers who generally have more helpers (i.e. who have a higher mean (*µ*) helper number) only have broods that experience higher provisioning rates to the extent that one would expect *solely* on the basis of the within-mother effect of helper numbers (i.e. the effect of *Δ* helper number).

We suggest that the most parsimonious explanation for these matching within-mother and population-level relationships between helper number and brood provisioning rate is that they reflect causal effects of helping behaviour. Helpers are known to contribute to offspring feeding [49,51], temporary helper removals reduce provisioning rates [51], and the observed relationship between helper number and provisioning rate cannot be readily attributed to the following alternative explanations either. The relationship cannot be attributed to mothers provisioning at higher rates when helped, because mothers significantly reduce their nestling provisioning rates when helped [53]. Nor can it be attributed to correlated variation in the mother's age, as maternal age is not correlated with helper numbers in this system [66]. Variation in helper numbers is also unlikely to be confounded by variation in abiotic environmental conditions, as (i) helper numbers do not fluctuate with short-term variation in environmental conditions [53] (typically being surviving young that remain in their natal group until death or dispersal [50]), (ii) our helper number measure excludes individuals younger than six months of age that may have survived to recruit *because* of recent rainfall conditions, and (iii) our analyses allow for effects of rainfall (the principal abiotic driver of productivity in this system [51,52]).

A direct effect of postnatal feeding by helpers would therefore also seem the most likely mechanism driving the observed relationships between helper number and offspring growth and telomere dynamics. Without cross-fostering eggs between groups with differing helper numbers though [16,48], it is impossible to rule out a role for indirect maternally mediated effects of helper numbers on prenatal investment in the egg (i.e. mothers modifying egg constituents according to helper numbers [48,53,83]). For example, sparrow-weaver mothers that have more helpers do lay slightly larger and heavier eggs, which yield slightly heavier hatchlings [53]. However, our follow-up analyses here confirmed that (i) the interaction between helper number and developmental rainfall in the nestling mass model is robust to allowing for an independent effect of egg volume (which positively predicted nestling mass), and (ii) there is no detectable effect of egg volume (or helper number) on telomere length soon after hatching (i.e. following the culmination of prenatal development). As such, the observed relationships between helper number and offspring traits cannot be readily attributed to correlated variation in overall maternal investment in the egg. It nevertheless remains conceivable that effects of helpers on postnatal growth and telomere dynamics arise, at least in part, via helper-driven changes in maternal prenatal deposition of critical egg constituents (such as hormonal signals or antioxidants; themselves a likely co-adaptation to the anticipated contributions of helpers [53,83,84]), given that this could occur independent of egg volume and have effects that only manifest during postnatal development [84].

### (b) Interactive effects of helpers and rainfall on offspring growth

Our analyses of offspring mass at 8 days of age suggest that helpers enhance nestling growth. The higher masses of helped nestlings (particularly in dry conditions) seem likely to yield an early-life benefit by reducing starvation risk; nestling mass is a positive predictor of survival to fledging in this species [37]. Any such positive effect of helping on offspring survival arising from effects on growth seems likely to be at its strongest in dry conditions, because the helper effect on growth is largest in dry conditions (when offspring are being fed at lower rates) and because the risk of nestling starvation is highest in dry conditions [51]. Indeed, our growth findings align well with, and could be one mechanism driving, our previous finding that helpers have their strongest positive relationship with offspring survival to fledging in dry conditions [51]. Whether any helper effect on offspring growth is likely to have lasting effects on offspring *beyond* fledging is less clear. Notably, helper numbers no longer positively predict offspring body mass or skeletal size following fledging; positive effects of rainfall dominate here (owing perhaps to rainfall impacting the nature of the food available, reflected by its positive effect on the size of prey delivered). The disappearance of this helper effect over time could reflect initially lighter offspring subsequently growing for longer or faster to catch up any deficit in size [57,58,85]. If initially lighter offspring then grew for longer, this could increase the risk of in-nest mortality by delaying fledging [57,58] but entail no further fitness cost beyond fledging. In this scenario, any benefits arising from a helper effect on early growth could be restricted to the nestling phase. However, if initially lighter offspring subsequently grew faster, such catch-up growth could entail costs to somatic maintenance and thus downstream performance in adulthood [57,58,85]. Indeed, such catch-up growth could be one mechanism contributing to the higher telomere attrition rates among offspring reared by fewer helpers [10,85]. In this scenario, offspring could conceivably gain lifelong benefits from helpers buffering early growth and thereby obviating the need for catch-up growth and its attendant downstream costs [57]. While the lack of detectable helper effects on fledgling morphology could solely reflect such compensatory changes in growth, it could also arise in part from selective mortality of lighter nestlings [37], which could leave the worst-affected nestlings (those born in dry conditions with few helpers) under-represented among the fledglings studied here.

The global association between cooperative breeding in birds and highly variable rainfall regimes [79–82] has led to the hypothesis that helping may yield particular benefits in such regimes by buffering the fitness components of recipients against fluctuations in rainfall [47,51,86]. While an increasing number of studies have now tested for such buffering effects of helpers, evidence of such effects remains rare [47,51,86]. Our finding that helpers appear to buffer offspring growth against variation in rainfall (i.e. reduce rainfall-induced variance in growth) is notable in this regard and accords with our previous work showing that sparrow-weaver helpers also appear to buffer offspring survival against variation in rainfall [51]. Positive effects of helpers on offspring survival in dry conditions (when food is scarce) are counteracted by negative effects of helpers in wet conditions (owing perhaps to helpers increasing the risk of nest predation), such that once one integrates across rainfall conditions, helpers reduce rainfall-induced *variance* in offspring survival (which may enhance breeder fitness via bet-hedging mechanisms [51]) but have no overall effect on the *average* rate of offspring survival to fledging [51]. Our second key finding, then, that helpers do appear to reduce the developmental telomere attrition rate, is notable then, as it suggests that while helpers may not improve the average rate of offspring survival to fledging, they may instead improve the downstream performance of those offspring that do survive to fledging.

### (c) Effects of helpers on developmental telomere attrition

The positive relationship between helper number and the within-individual rate of change in telomere length during development implicates a role for helping in bolstering the somatic integrity of offspring. This finding cannot be readily attributed to several alternative explanations. First, it cannot be attributed instead to beneficial effects of the offspring foraging in a larger group (independent of the receipt of help), as (i) while the latest telomere measures in the time series were collected from young fledglings, offspring rarely forage with their groups at this age (instead remaining hidden in or near the nest tree), (ii) previous findings suggest that subordinate birds living in larger groups actually suffer reduced rather than enhanced foraging success (as they show poorer body condition and lower survival rates [56]), and (iii) previous analyses have not detected beneficial effects of group size on telomere attrition in adulthood [87]. Second, it cannot be readily attributed to correlated changes in prenatal development (e.g. owing to maternal plasticity in egg investment [53]) leaving offspring with more helpers already differing in telomere lengths *before* the helping period, as the telomere lengths of recently hatched chicks (the products of prenatal development) were unrelated to both egg volume and helper numbers. Third, it is unlikely that the relationship between helper number and telomere attrition arises via correlated changes in clutch or brood size (which could alter telomere dynamics via effects on the intensity of resource competition [10,12,13]), because sparrow-weaver mothers do not alter clutch size with helper number [53] and our analyses allow for effects of brood size. Finally, it is unlikely that this effect is driven instead by correlated variation in parental age (which can itself influence early-life telomere dynamics [44,66]). While we have detected positive effects of within-parent variation in age on offspring telomere length in this species ([66] maternal and paternal age are correlated, leaving their effects difficult to tease apart), (i) maternal age is unrelated to helper numbers [66], and (ii) paternal age is, if anything, *negatively* correlated with helper numbers [66] (such that any beneficial effects of advanced paternal age would be expected to manifest in groups with *fewer* helpers and thus counteract rather than confound the relationship observed here).

As such, perhaps the most plausible explanation for the positive relationship between helper numbers and the within-individual rate of change in telomere length is that the positive effect of helper number on provisioning rate allows offspring to invest more heavily in somatic maintenance. Such an effect could arise via a range of mechanisms, including helpers (i) increasing the resources available for somatic maintenance (e.g. to mitigate oxidative stress and/or repair oxidative damage [23,24]), (ii) ameliorating any associated stress responses (given the potential for resource shortages to elevate glucocorticoid levels [88] and for stressor exposure to accelerate telomere attrition [34,45]), and (iii) reducing the need for concurrent costly investments in competitive activities such as begging [14]. Our growth analyses highlight the possibility that helpers also reduce nestling telomere attrition in part via their effects on growth, independent of any effect that they may also have on resource availability; unhelped offspring may engage in ‘catch-up’ growth acceleration during late development to attain a comparable size at fledging (see above), and such growth acceleration has the potential to accelerate telomere erosion [23,59]. Helpers might thereby reduce the developmental telomere attrition rate in part by stabilizing offspring growth, obviating the need for such ‘catch-up’ growth and its potential attendance costs.

As telomere attrition is thought to serve as a biomarker of the accumulation of somatic deficits (see ‘Introduction’), a positive effect of helping on the rate of change in telomere length during development suggests that helpers could have cryptic beneficial effects on offspring somatic integrity, with the potential to impact their downstream survival, performance and ageing trajectories. This possibility is rendered credible for sparrow weavers by our previous finding that the within-individual rate of change in telomere length during development (the same metric that helper numbers positively predict here) positively predicts downstream survival to adulthood, while telomere length *per se* does not [37] (see [10,40] for similar findings in Eurasian jackdaws, *Corvus monedula*, and zebra finches, *Taeniopygia castanotis*; though also see [41,42]). These findings align with expectation if the telomere attrition rate acts as a biomarker of the accumulation of correlated damage to other structures whose deficits are more causal than telomere length in undermining tissue performance [10,37]. They also underscore the potential for helper effects on developmental telomere attrition to have downstream fitness consequences for offspring, even in the absence of a detectable effect (in our analyses) of helpers on telomere length at fledging.

The lack of a detectable effect of helpers on telomere length at fledging cannot be readily attributed to a pre-existing positive effect of helpers on telomere length post-hatching (which a helper effect on postnatal telomere attrition might then offset), as helper numbers did not predict telomere length soon after hatching or in the first telomere length measures available for the nestlings that contributed to our telomere erosion analysis (see ‘Results’). Conceivably, selective disappearance of nestlings with high telomere attrition rates (given the link between telomere attrition rate and offspring mortality in this species [37]) could have reduced the representation of the helper effect on telomere attrition among those offspring still available for resampling at fledging. It is also possible that limited statistical power in our fledgling analysis hampered the detection of a helper effect. Perhaps most plausibly though, any helper effect on postnatal telomere attrition may be being swamped at fledging by pre-existing inter-individual variation in mean telomere length (repeatable in this species [87]). Were such additional sources of inter-individual variation themselves reflective of variation in somatic integrity, it would be reasonable to conclude that the helper effect alone may have little impact on the somatic integrity (and thus downstream performance) of offspring. However, our prior finding that inter-individual variation in telomere attrition rate predicts offspring survival to adulthood in this system while inter-individual variation in telomere length does not [37] gives cause to question the biological relevance of the latter in this system, given its potential to arise via a range of mechanisms unrelated to somatic integrity. For example, it has been suggested that genetic and/or epigenetic variation in a notional ‘initial’ telomere length set during early development could be unrelated to somatic integrity ([10,37,43,44]; see also [41,42]). Furthermore, in our studies, the use of qPCR to estimate mean telomere lengths could also introduce artefactual inter-individual variation in mean telomere length estimates, as this method estimates the abundance of telomeric DNA sequence repeats across the genome, capturing not only those that lie within terminal telomeres but also those that lie within interstitial chromosomal regions [89] (whose abundance is not generally expected to be temporally dynamic or to have functional consequences). While our analyses of the effects of helper number on the within-individual rates of change in telomere length should be robust to any such artefactual source of consistent inter-individual differences in apparent telomere length, our estimates of telomere length *per se* at fledging would not. It is therefore conceivable that the true mean telomere lengths of sparrow-weaver fledglings actually are positively affected by helper numbers (owing to helper-driven amelioration of telomere attrition), but that this effect is being shrouded in our analyses by artefactual inter-individual variation in length estimates.

Our telomere findings provide a rare example of effects of cooperative interactions on telomere attrition rates [46] and highlight that such effects could be more widespread among cooperative breeders. Such effects could provide a general explanation (alongside helper effects on adult body size or condition) for beneficial downstream effects of helping on offspring performance in adulthood [1,2,7,26] and highlight the potential for helpers to shape the downstream senescence trajectories of offspring (as they may also do for parents [46]). Notably though, studies of at least four cooperative breeders have not yielded clear evidence of helper effects on offspring telomere length [13,16–18] (see ‘Introduction’). That the relationship between helper number and telomere attrition in our study was not detectable in the telomere lengths of fledglings highlights the possibility that helper effects could have gone undetected in previous studies owing to a focus on telomere lengths rather than within-individual rates of telomere erosion [13,16–18]. That said, it is also likely that cooperative breeders vary in the extent to which additive effects of helpers on provisioning lead to improvements in the somatic integrity of offspring. For example, in some cooperative breeders, mothers may compensate for helper contributions by reducing their own investment in the egg, a mechanism that might account for the lack of a detectable effect of helpers on offspring telomere lengths in superb fairy wrens [18,48]. Species may also differ in the extent to which selection favours offspring investing helper-derived resources into growth versus self-maintenance, owing, for example, to interspecific differences in the pay-offs to be had from a large body size versus a longer potential lifespan. Indeed, our detection of helper effects on telomere attrition but not fledgling size could reflect the fact that the primary determinant of winning a rare breeding vacancy in sparrow weavers is not large size but old age: vacancies are taken by the oldest birds in the area [90]. Cooperative species could also differ in the extent to which helpers impact the telomeres of offspring versus parents, owing to interspecific variation in the extent to which parents reduce their provisioning effort when helped [21] (a mechanism by which helpers could reduce telomere attrition among parents [46]). As sparrow-weaver helpers do reduce the provisioning workloads of mothers [53], leading to reduced oxidative stress and improved maternal survival [56,63,91], helping in our study system might ultimately prove to benefit the telomere dynamics of mothers as well as offspring.

### (d) Summary and wider implications

Our findings provide rare evidence that cooperative behaviour can reduce developmental telomere attrition and buffer offspring growth against unpredictable rainfall [47,51,86], shedding new light on the mechanisms by which helpers may impact the fitness of recipients, and the reasons why avian cooperative breeding is globally associated with unpredictable rainfall (see above). Our findings have wider implications too for understanding the resilience of cooperative breeders to anthropogenically induced global change. Southern Africa, and the Kalahari region in particular, is predicted to experience reduced annual rainfall and a growing incidence of drought over the coming years [92]. As rainfall is the primary driver of productivity in this landscape, the biotic consequences of these changes in rainfall could be marked. In white-browed sparrow-weaver societies, rainfall is the major trigger for breeding and the principal driver of offspring survival [51,52]. Our finding that developmental rainfall also positively predicts fledgling mass and size highlights the potential for offspring reared in drought conditions to suffer lagged lifelong costs arising from their hard start. On the positive side, our findings also highlight the scope for helpers to buffer offspring growth against low rainfall (just as our previous work has shown for offspring survival [51]) and complement similar evidence of buffering in two other cooperative breeders from the Kalahari [47,86]. While social buffering could leave high-density populations (with higher helper numbers) more robust to changing rainfall patterns, they could also leave populations vulnerable to novel Allee effects on their resilience [93]: if a population crash were to reduce helper numbers, this would undermine helper-mediated buffering in any ensuing drought years. Attempts to predict the plight of social species across the global drylands will therefore benefit from studying how social and abiotic effects interact to shape development, somatic integrity and performance [17,18,94].

## Supplementary Material



## Data Availability

All R scripts and datasets needed to reproduce the analyses presented in this paper are available at https://github.com/PabloCapilla/cooperation_development [95]. Supplementary material is available online [96].

## Theme

One contribution of 21 to a theme issue ‘Developmental circuits in ageing’.
