# Supplementary material for: Cooperative care slows offspring telomere attrition and buffers growth against variable rainfall in a wild Kalahari bird
Source: Philos Trans R Soc Lond B Biol Sci. 2026 Nov 12;381(1963):20250325. doi: 10.1098/rstb.2025.0325 (PMC13755915; doi:10.1098/rstb.2025.0325)
Supplement: Supplementary file 1 [file rstb.2025.0325.zip › rstb.2025.0325/rstb20250325_si_001.docx]

**Supplementary Materials for:**

**Cooperative alloparental care slows offspring telomere attrition
and buffers growth against variable rainfall in a wild Kalahari bird**

Pablo Capilla-Lasheras^1,2,3,4*^, Emma M. Wood^1*^, Antony M. Brown^1^, Andrew J. Young^1∇^

^1^ Centre for Ecology and Conservation, University of Exeter Cornwall Campus, Penryn, Cornwall TR10 9EZ, UK

^2^ Swiss Ornithological Institute, Bird Migration Unit, Sempach, Switzerland

^3^ Doñana Biological Station, Spanish National Research Council (EBD-CSIC), Sevilla, Spain

^4^ School of Biodiversity, One Health and Veterinary Medicine, University of Glasgow, Glasgow, UK

^*^ Joint first authors

^∇^ Corresponding author

[A.J.Young@exeter.ac.uk](mailto:A.J.Young@exeter.ac.uk)
Centre for Ecology and Conservation

University of Exeter, Cornwall Campus

Tremough, Cornwall TR10 9EZ

UK

**SUPPLEMENTARY TABLES**

**Table S1 – Factors affecting nestling mass (at 7-9 days of age) allowing for an effect of egg volume.** Model estimates, standard errors (SE), and their 95% confidence intervals (95% CI) are provided along with results from likelihood-ratio tests (χ^2^) assessing the statistical significance of each predictor within the full model (i.e., a model containing all terms in the table below). N = 408 nestling mass measurements collected across 9 seasons from 35 social groups rearing 261 clutches. Random effect standard deviations (in g): 'clutch ID' = 2.50, 'group ID' = 0.00, 'mother ID' = 0.73, 'season' = 1.49. Developmental rainfall and egg volume were mean centred and scaled by one standard deviation prior to model fitting to improve model convergence.

| **Fixed effect** | **Estimate** | **SE** | **95% CI** | **χ^2^** | **df** | **p-value** |  |
| --- | --- | --- | --- | --- | --- | --- | --- |
| Intercept | 18.65 | 1.16 | 16.37, 20.92 |  |  |  |  |
| Developmental rainfall (mm) | 1.88 | 0.40 | 1.09, 2.67 |  |  |  |  |
| Helper number | 0.20 | 0.15 | -0.09, 0.49 |  |  |  |  |
| Age (days) |  |  |  | 79.33 | 2 | **< 0.001** |  |
| *7* | — | — | — |  |  |  |  |
| *8* | 2.75 | 0.58 | 1.61, 3.89 |  |  |  |  |
| *9* | 5.66 | 0.62 | 4.45, 6.87 |  |  |  |  |
| Brood size | -0.49 | 0.42 | -1.31, 0.34 | 1.27 | 1 | 0.259 |  |
| Egg volume (mm³) | 0.92 | 0.24 | 0.45, 1.38 | 14.32 | 1 | **< 0.001** |  |
| Developmental rainfall (mm) × Helper number | -0.26 | 0.12 | -0.49, -0.03 | 4.88 | 1 | **0.027** |  |
| Abbreviations: CI = Confidence Interval, SE = Standard Error, df = degrees of freedom. | | | | | | | |

**Table S2 – Factors affecting fledgling tarsus length at 30-45 days of age.** Model estimates, standard errors (SE), and their 95% confidence intervals (95% CI) are provided along with results from likelihood-ratio tests (χ^2^) assessing the statistical significance of each predictor within the full model (i.e., a model containing all terms in the table below). N = 132 fledgling tarsus length measurements collected across 9 seasons from 34 social groups rearing 102 clutches. Random effect standard deviations (in mm): 'clutch ID' = 0.12, 'group ID' = 0.00, 'mother ID' = 0.28, 'season' = 0.34. Developmental rainfall was mean centred and scaled by one standard deviation prior to model fitting to improve model convergence. The interaction between helper number and developmental rainfall did not significantly explain variation in fledgling tarsus length (χ^2^_1_ = 0.16, p = 0.693).

| **Fixed effect** | **Estimate** | **SE** | **95% CI** | **χ^2^** | **df** | **p-value** |
| --- | --- | --- | --- | --- | --- | --- |
| Intercept | 24.08 | 0.49 | 23.11, 25.05 |  |  |  |
| Developmental rainfall (mm) | 0.14 | 0.07 | 0.01, 0.27 | 4.21 | 1 | **0.040** |
| Helper number | 0.01 | 0.04 | -0.07, 0.10 | 0.04 | 1 | 0.851 |
| Age (days) | 0.01 | 0.01 | -0.02, 0.03 | 0.42 | 1 | 0.516 |
| Brood size | -0.15 | 0.09 | -0.33, 0.04 | 2.49 | 1 | 0.115 |
| Sex |  |  |  | 10.56 | 2 | **0.005** |
| *Female* | — | — | — |  |  |  |
| *Male* | 0.33 | 0.10 | 0.14, 0.52 |  |  |  |
| *Unknown* | 0.07 | 0.23 | -0.39, 0.52 |  |  |  |
| Abbreviations: CI = Confidence Interval, SE = Standard Error, df = degrees of freedom. | | | | | | |

**Table S3 – Factors affecting fledgling wing length at 30-45 days of age.** Model estimates, standard errors (SE), and their 95% confidence intervals (95% CI) are provided along with results from likelihood-ratio tests (χ^2^) assessing the statistical significance of each predictor within the full model (i.e., a model containing all terms in the table below). N = 133 fledgling wing length measurements collected across 9 seasons from 34 social groups rearing 103 clutches. Random effect standard deviations (in mm): 'clutch ID' = 1.95, 'group ID' = 0.00, 'mother ID' = 0.00, 'season' = 1.27. Developmental rainfall was mean centred and scaled by one standard deviation prior to model fitting to improve model convergence. The interaction between helper number and developmental rainfall did not significantly explain variation in fledgling mass (χ^2^_1_ = 0.18, p = 0.673).

| **Fixed effect** | **Estimate** | **SE** | **95% CI** | **χ^2^** | **df** | **p-value** |  |
| --- | --- | --- | --- | --- | --- | --- | --- |
| Intercept | 88.05 | 3.19 | 81.80, 94.31 |  |  |  |  |
| Developmental rainfall (mm) | 0.70 | 0.43 | -0.14, 1.54 | 2.95 | 1 | 0.086 |  |
| Helper number | -0.08 | 0.27 | -0.61, 0.45 | 0.15 | 1 | 0.701 |  |
| Age (days) | 0.31 | 0.08 | 0.15, 0.47 | 15.10 | 1 | **< 0.001** |  |
| Brood size | -0.82 | 0.66 | -2.11, 0.47 | 1.45 | 1 | 0.229 |  |
| Sex |  |  |  | 3.34 | 2 | 0.189 |  |
| *Female* | — | — | — |  |  |  |  |
| *Male* | 1.30 | 0.69 | -0.06, 2.65 |  |  |  |  |
| *Unknown* | 0.94 | 1.64 | -2.27, 4.15 |  |  |  |  |
| Abbreviations: CI = Confidence Interval, SE = Standard Error. df = degrees of freedom | | | | | | | |

**Table S4 – Factors affecting nestling mean whole blood telomere length soon after hatching (less than 7 days of age) allowing for an effect of egg volume.** Model estimates, standard errors (SE), and their 95% confidence intervals (95% CI) are provided along with results from likelihood-ratio tests (χ^2^) assessing the statistical significance of each predictor within the full model (i.e., a model containing all terms in the table below). N = 69 telomere length estimates collected across 5 seasons from 28 social groups rearing 48 clutches. Random effect standard deviations: 'clutch ID' = 0.12, 'group ID' = 0.00, 'mother ID' = 0.00, 'season' = 0.09, 'PCR plate ID' = 0.08. Developmental rainfall and egg volume were mean centred and scaled by one standard deviation prior to model fitting to improve model convergence.

| **Fixed effect** | **Estimate** | **SE** | **95% CI** | **χ^2^** | **df** | **p-value** |  |
| --- | --- | --- | --- | --- | --- | --- | --- |
| Intercept | 1.45 | 0.31 | 0.85, 2.05 |  |  |  |  |
| Developmental rainfall (mm) | 0.04 | 0.04 | -0.04, 0.12 | 1.13 | 1 | 0.287 |  |
| Helper number | 0.01 | 0.02 | -0.03, 0.04 | 0.24 | 1 | 0.625 |  |
| Age (days) | -0.08 | 0.05 | -0.16, 0.01 | 2.35 | 1 | 0.125 |  |
| Mean egg volume (mm³) | -0.04 | 0.03 | -0.10, 0.02 | 2.70 | 1 | 0.101 |  |
| Storage (days) | 0.08 | 0.08 | -0.07, 0.23 | 1.23 | 1 | 0.268 |  |
| Brood size | -0.01 | 0.05 | -0.10, 0.09 | 0.04 | 1 | 0.834 |  |
| Sex |  |  |  | 0.09 | 1 | 0.761 |  |
| *Female* | — | — | — |  |  |  |  |
| *Male* | 0.00 | 0.05 | -0.11, 0.10 |  |  |  |  |
| Abbreviations: CI = Confidence Interval, SE = Standard Error, df = degrees of freedom | | | | | | | |
